# Non-Invasive Retinal Imaging Modalities for the Identification of Prognostic Factors in Vitreoretinal Surgery for Full-Thickness Macular Holes

**DOI:** 10.3390/diagnostics13040589

**Published:** 2023-02-05

**Authors:** Cristina Nicolosi, Giulio Vicini, Daniela Bacherini, Dario Giattini, Noemi Lombardi, Claudio Esposito, Stanislao Rizzo, Fabrizio Giansanti

**Affiliations:** 1 Eye Clinic, Neuromuscular and Sense Organs Department, Careggi University Hospital, 50134 Florence, Italy; 2 Department of Neurosciences, Psychology, Drug Research and Child Health, University of Florence, 50121 Florence, Italy; 3 Azienda USL Toscana Nord Ovest, 56121 Pisa, Italy; 4 Ophthalmology Unit, Catholic University of the Sacred Heart, Fondazione Policlinico Universitario A. Gemelli, 00168 Rome, Italy; 5 Consiglio Nazionale delle Ricerche (CNR), 56124 Pisa, Italy

**Keywords:** retinal imaging, full-thickness macular hole, prognostic factors, biomarkers, OCT, OCTA

## Abstract

In this review, we will focus on different non-invasive retinal imaging techniques that can be used to evaluate morphological and functional features in full-thickness macular holes with a prognostic purpose. Technological innovations and developments in recent years have increased the knowledge of vitreoretinal interface pathologies by identifying potential biomarkers useful for surgical outcomes prediction. Despite a successful surgery of full-thickness macular holes, the visual outcomes are often puzzling, so the study and the identification of prognostic factors is a current topic of interest. Our review aims to provide an overview of the current knowledge on prognostic biomarkers identified in full-thickness macular holes by means of different retinal imaging tools, such as optical coherence tomography, optical coherence tomography angiography, microperimetry, fundus autofluorescence, and adaptive optics.

## 1. Introduction

Idiopathic macular hole, also known as full-thickness macular hole (FTMH), is a foveal defect involving all the neuroretinal layers, from the internal limiting membrane (ILM) to the photoreceptor layer [1]. Idiopathic FTMH affects 1 in 250 people and results in significant visual impairment, with central visual loss and metamorphopsia [2]. The pathogenesis of the disease is a result of an anomalous posterior vitreous detachment leading to pathological anteroposterior traction of the vitreous cortex on the fovea and perifoveal area [3]. Also, tangential tractional forces may play a role in the enlargement of the hole and in the elevation of its edges, and disruption and loss of function of foveal Müller cells seem to be involved in the FTMH formation process [4].

The most accepted classification is the OCT-based staging proposed by the International Vitreomacular Traction Study (IVTS) group in 2013 [5], which updated the previous ophthalmoscopic classification by Gass [1]. In this classification, FTMHs are associated (early stage) or not (end-stage) with a vitreomacular traction (VMT) derived from a vitreomacular adhesion (VMA). FTMHs are classified into three subgroups based on the horizontal linear width at the narrowest point of the hole: small (≤250 μm), medium (>250 μm and ≤400 μm), and large (>400 μm). Recently, some authors discussed the possibility of defining a FTMH large when the minimum linear width was greater than 650 μm, after clinical observations of a highly successful rate of closure after surgery in patients affected by FTMH less than 650 μm in width [6].

Pars plana vitrectomy (PPV) with gas or air tamponade is widely accepted as the gold standard technique, with reported closure rates after the first surgery ranging from 78 to 96% [7,8,9,10]. However, a successful anatomical closure after surgery does not necessarily correspond to visual improvement, and the visual outcomes are often puzzling. In a recent study, the visual acuity (VA) improvement was less than 15 Early Treatment of Diabetic Retinopathy Study (ETDRS) letters at 24 months in a third of patients successfully operated for FTMH, and the final VA was less than 70 ETDRS letters [11].

The technological innovations and developments in retinal multimodal imaging made in recent years have increased the knowledge of FTHMs by identifying a series of potential biomarkers useful for surgical outcomes prediction. In addition to widely accepted prognostic factors of FTHM, such as stage and size, symptoms duration, and preoperative VA [12,13,14], several studies have recently explored new preoperative predictors for visual outcomes after PPV for FTMH, both morphological and functional, by means of different non-invasive imaging modalities.

In this review, we will focus on non-invasive retinal imaging techniques that can be used to assess morphological and functional features in FTMH, evaluating the contribution of each imaging modality in the identification of prognostic factors of visual outcome. Our review aims to provide an overview of different non-invasive retinal imaging tools in FTMH and the prognostic significance of their findings.

## 2. Non-Invasive Retinal Imaging in Vitreoretinal Interface Diseases

The integration of several non-invasive imaging techniques has recently gained importance in the characterization of vitreomacular interface pathologies, including FTMHs. Multimodal imaging allows the integration of images acquired with multiple modalities, such as optical coherence tomography (OCT), OCT angiography (OCTA), fundus autofluorescence (FAF), fundus photography, and adaptive optics (AO). Functional imaging modalities, such as microperimetry (MP), can assess the retinal sensitivity and add measurable parameters of visual outcome. The aim of integrated imaging is to overcome the limitations of a single technique obtaining a more thorough comprehension of the morphological and functional features of the disease.

The role of different retinal imaging modalities in the evaluation of FTMHs is summarized in Table 1.

Table 2 summarizes key findings in FTMHs prognostic evaluation by means of different retinal imaging modalities.

### 2.1. Structural Optical Coherence Tomography (OCT)

**Figure 1:**
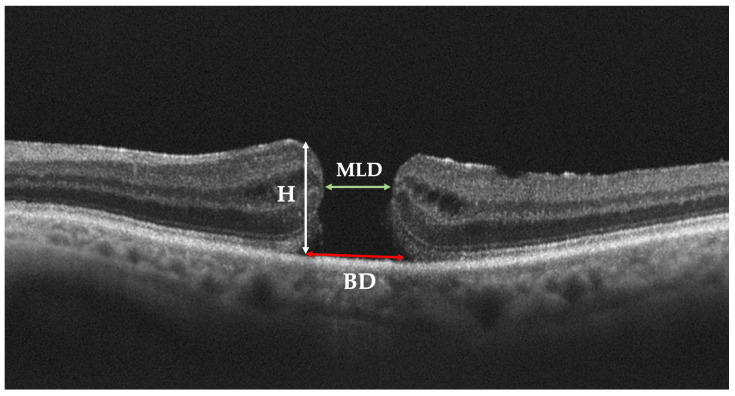
Preoperative parameters of full-thickness macular hole (FTMH) on Optical Coherence Tomography (OCT) scan. Minimum linear diameter (MLD), base diameter (BD), and hole height (H) are shown.

Structural OCT imaging plays an essential role in diagnosing and managing FTMHs. OCT has traditionally enabled an accurate diagnosis and differentiation of vitreomacular pathologies and a potential prediction of visual prognosis after successful surgery. In patients with FTMH, structural OCT has been first used to measure the size of the hole, drawing with the caliper function a horizontal line connecting the two closest components of the retina (minimum linear dimension) (Figure 1). Other measurements, such as the base diameter and the height of the hole, have also been studied. The macular hole index (height/maximum basal diameter), a ratio easily calculated from OCT images of the macular area, represents the preoperative configuration of a macular hole and is a prognostic factor for visual outcome [15].

The minimum linear dimension is a critical factor in determining the anatomical success rate of hole closure after surgery, and is the basis for the international OCT-based classification system for FTMH. The success of the surgery and the postoperative VA is better when the hole diameter is smaller in size [5,12,15,16,17,18,19].

Recently, other OCT structural features as predictors of functional recovery after surgery for FTMH have been investigated.

We will discuss the following preoperative OCT biomarkers: external retinal layers status, intraretinal cystic changes, supra-retinal pigment epithelium (RPE) granular deposits, macular holes borders morphology, and epiretinal proliferation.

**Figure 2:**
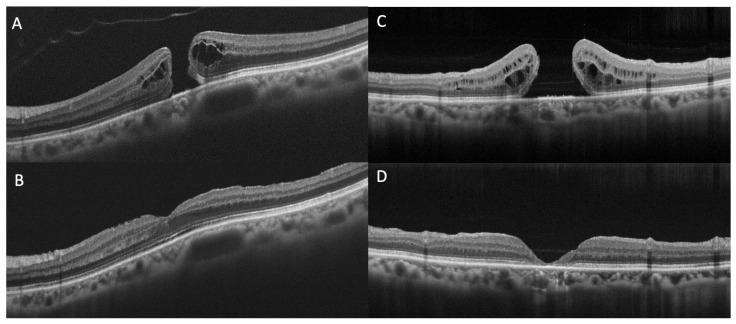
Optical Coherence Tomography (OCT) scans of full-thickness macular holes and defects in external limiting membrane (ELM), ellipsoid zone (EZ), and interdigitation zone (IZ) at baseline (**A**,**C**) and at six months follow-up (**B**,**D**). (**A**) Preoperative ELM, EZ and IZ are almost preserved and at postoperative follow-up (**B**) a quite full subfoveal restoration of the three bands can be appreciated. (**C**) Preoperative ELM, EZ and IZ defects are extensive and at postoperative follow-up (**D**) subfoveal atrophy development can be observed.

Concerning the external retinal layers, structural OCT allows the delineation of three hyperreflective bands: the external limiting membrane (ELM), the ellipsoid zone (EZ), and the interdigitation zone (IZ) [20]. The EZ and IZ were previously known as the junction of the inner and outer photoreceptor segments (inner segment (IS)/ outer segment (OS) junction) and the cone outer segment tips line, respectively [21]. Numerous works have investigated the correlation between preoperative defects and/or postoperative remodeling of the ELM, EZ, and IZ and VA in patients with FTMHs (Figure 2) [22,23,24,25]. There is a consensus in the literature that the restoration of these three bands is correlated with VA [26,27]. Houly et al. quantitatively evaluated the correlation between the length of preoperative defects of the three bands and VA at 3 and 6 months after surgery, finding a significant association [28]. In particular, the preoperative length of the ELM defect was found as the strongest predictor of VA after FTMH surgery.

**Figure 3:**
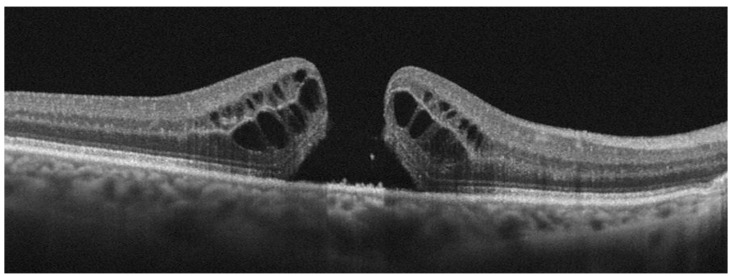
Optical Coherence Tomography (OCT) scan of a full-thickness macular hole associated with intraretinal cystoid spaces. Multiple hyporeflective cystoid spaces are located in both the inner nuclear layer and the outer nuclear layer.

It has been reported that idiopathic FTMHs may be associated with cystic changes. Intraretinal cystoid cavities at the edges of an FTMH have been recognized in the earliest OCT images as intraretinal hyporeflective spaces, frequently observed around the edges of FTMHs (Figure 3) [29]. It is believed that the cystoid spaces expand and separates the retinal layer along with FTMH forming. Moreover, the intraretinal cystoid cavities have been observed around the edges of the FTMH [30]. Different studies have been conducted on the correlation between the presence of cystoid cavities and postoperative visual outcomes, but they showed contrasting results. The presence of intraretinal cysts seems to be positively related to the diameter and height of the FTMH [31,32].

Ozturk et al. quantified the cyst and macular hole areas, finding a moderate negative correlation with postoperative best-corrected visual acuity (BCVA) [31]. Roth et al. found that the areas of parafoveal intraretinal pseudocysts were correlated with a low closure rate and with low postoperative VA [19], accordingly to Ruiz-Moreno et al. findings, reporting that the mean pre- and postoperative VA was lower in patients with cystic retinal changes than in those without [33]. In contrast, Brockmann et al. found that the presence of parafoveal cysts was associated with a higher closure rate, but in their work, the cysts were only assessed qualitatively [34]. In a similar approach by Chhablani et al., the presence of cystic edges was associated with anatomical success and a better final VA [35]. In some studies, the cystoid cavities seemed to be not significantly correlated with postoperative BCVA, but negatively correlated with the preoperative BCVA and with the degree of postoperative metamorphopsia [36,37]. Joo et al. hypothesized that intraretinal cysts in FTMH may help to predict hole closure but also damage to photoreceptors that affects postoperative visual prognosis [38]. They hypothesized that cystic changes may affect VA negatively by increasing the height of the hole edges. However, they found that the surgical outcome improved as the size of the cysts increased, so they speculated that the cystic changes may correlate with good VA after surgery if significant functional retinal tissues persist after closure. In a recent report by Govetto et al., cystoid spaces have been characterized by means of multimodal imaging (OCT, OCTA, blue-FAF, and fluorescein angiography) in both exudative and tractional macular disorders, including idiopathic FTMHs, in order to make physiopathological considerations [39]. The multimodal imaging in FTMH identified features of both exudative and tractional cystoid spaces without any leakage or pooling in fluorescein angiography. The cystic spaces displayed a characteristic “sunflower” appearance on en face OCT, with radial hyporeflective spaces radiating from a central hyporeflective area. The authors proposed a pathophysiological mechanism for the origin of cystic changes in FTMH, due to RPE contact loss at the edge of the hole. In the detached neuroretina, the outward-directed RPE pump function is reduced. It does not effectively compensate the inward osmotic gradient between the photoreceptors layer and the ILM, favoring the flow of interstitial fluid through the retina [40] and the formation of cystoid spaces at the edge of the hole without any vascular disruption. The enlargement of the cystoid spaces due to the inflow of fluid may produce an exudative-like morphology. This pathophysiological mechanism is also supported by Nair et al., who correlated the cystic area dimension in the outer and inner plexiform layers, along with their relationship to the basal diameter of FTMH [32].

Also, the pathophysiology of intraretinal cystoid spaces in FTMH may involve other pathways distinct from exudation and traction, including degenerative cellular loss and retrograde trans-synaptic degeneration, among others [41]. The prognostic significance of cystic changes in FTMH remains not fully understood.

**Figure 4:**
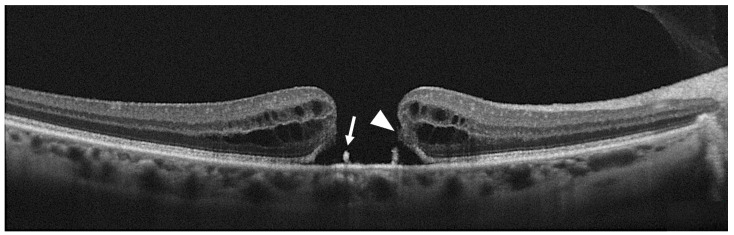
Optical Coherence Tomography (OCT) scan showing photoreceptor outer segment disruption at the macular hole borders (arrowhead). Supra−retinal pigment epithelium hyperreflective granular deposits are also visible at the base of the hole (arrow).

Another specific morphological characteristic of idiopathic FTMH is the presence of supra-RPE granular deposits (Figure 4), which has been recently investigated as a potential biomarker of negative prognosis. Govetto et al. conducted a retrospective, multicenter, interventional case series of 149 eyes of 143 consecutive patients diagnosed with FTMH, surgically treated, with a minimum follow-up of 12 months [42]. The authors found supra-RPE granular deposits in 81.2% of the eyes. They identified this characteristic as a significant predictor of lower postoperative BCVA, hypothesizing that it may be an indicator of photoreceptor disruption in FTMHs. They also distinguished between smooth borders (even and regular surface, free of perceptible projections, lumps, or indentations) and bumpy borders (uneven and irregular surface, with evident lumps or indentations), finding a smooth morphology in 38.9%, and a bumpy border in 61.1%. A bumpy morphology may suggest deeper and potentially irreversible photoreceptor damage and may negatively influence both functional and anatomical recovery. The authors found a significant correlation between lower postoperative BCVA, lower BCVA gain, and poor postoperative anatomical restoration, hypothesizing an association with deeper and potentially irreversible photoreceptor damage that may negatively influence both functional and anatomical recovery.

**Figure 5:**
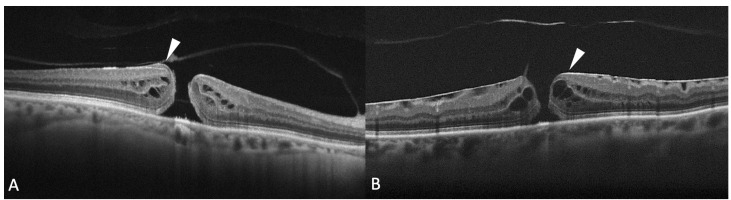
(**A**) Optical coherence tomography (OCT) scan showing a full-thickness macular hole (FTMH) accompanied by atypical epiretinal tissue (arrowhead); (**B**) OCT scans showing a hyperreflective epiretinal membrane associated with FTMH (arrowhead).

It has been reported that FTHM can be associated with the presence of epiretinal proliferation, also known as atypical epiretinal tissue, identified in structural OCT. Epiretinal proliferation presents as a premacular tissue with homogenous medium reflectivity over the internal limiting membrane on OCT and it is distinct from a hyperreflective tractional epiretinal membrane (Figure 5). Although it is most frequently associated with a lamellar hole, some cases of FTMH also show atypical epiretinal tissue at the edge of the hole. FTMHs with epiretinal proliferation have been reported to have worse clinical and surgical outcomes than FTMH without it [43]. The imaging and histopathological findings imply that the development of FTMH with epiretinal proliferation may not be reconducted to vitreomacular traction. Instead, FTMH with atypical epiretinal tissue might have evolved from lamellar holes with atypical epiretinal tissue. Bae et al. also explored the presence of atypical epiretinal tissue in FTMHs and its pathogenic and prognostic significance [44]. The authors studied 225 consecutive eyes of 211 patients who underwent surgery for an idiopathic FTMH. Eyes were divided into two groups according to the presence of epiretinal proliferation. It was found in 11.6% of the eyes. At baseline, eyes with atypical epiretinal tissue more frequently had a splitting of the inner retina but fewer intact photoreceptors compared with eyes without it. The presence of atypical epiretinal tissue was associated with moderately poorer outcomes at 12 months after surgical treatment, probably due to a large number of defects in the ELM, EZ, and IZ observed postoperatively. So, the authors hypothesized that the presence of epiretinal proliferation in an FTMH was related to poorer anatomical success and less visual recovery after surgery, suggesting that it reflects a chronic pathogenic process involving more severe damage to the foveal tissue. Ishida et al. evaluated the presence of preretinal abnormal tissue (atypical epiretinal tissue, perivascular glia, and a preretinal hyperreflective band) in a study on 60 eyes with FTMH, finding it in most eyes (94%) [45]. In 24%, the abnormal tissue was contiguous to the hole. In the others, it was extrafoveal. Eyes with preretinal tissue contiguous to the FTMH had worse baseline VA.

### 2.2. Fundus Microperimetry

**Figure 6:**
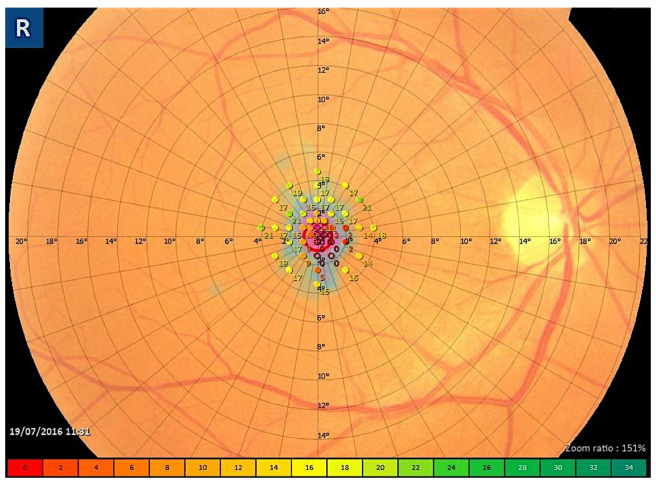
Microperimetric sensivity map in a case of full-thickness macular hole: an absolute central scotoma is evident with a surrounding ring of relative scotoma.

Fundus microperimetry (MP) is a precise, repeatable, non-invasive modality that provides a functional evaluation of retinal sensitivity (RS) and can be used as a complementary technique in addition to VA measurement to better assess changes in central macular function in patients with vitreomacular pathologies, including FTMHs [46,47,48]. Macular RS measured by MP rather than VA can better reflect the extent of visual function recovery after FTMH surgical repair [46,47]. Moreover, VA alone can underestimate the functional benefit of FTMH surgery [48]. The foveal sensitivity in FTMHs has also been evaluated in correlation with structural OCT, and parameters of vessel density evaluated with OCTA, as explained in the following paragraph. Figure 6 show retinal sensivity map obtained with microperimeter in a patient with FTMH.

### 2.3. Optical Coherence Tomography Angiography (OCTA)

OCTA is a non-invasive tool that allows the visualization and measurement of macular microcirculation without dye injection. It can produce high-resolution images of the retinal vasculature and is useful in the analysis of retinal disorders. With OCTA, it is possible to quantify the vessel density in the superficial and deep retinal capillary plexuses (SCP and DCP) and to evaluate the morphology of the foveal avascular zone (FAZ).

**Figure 7:**
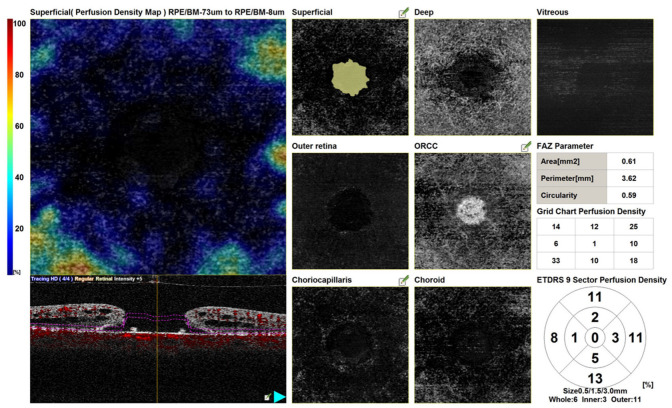
Optical coherence tomography angiography maps of a full-thickness macular hole.

Although several studies report no conclusive data, OCTA can be considered an objective and non-invasive tool for the evaluation and monitoring of the retinal microvascular changes and quantitative characteristics in FTMH before and after surgical treatment (Figure 7) [49,50,51,52,53,54,55,56].

The main issues in the OCTA study of FTMH are about the segmentation, which may produce image artifacts, with consequent difficulties in data interpretation [53]. For this reason, OCTA results should be carefully evaluated.

The main vascular alterations detected with OCTA in FTMHs seem to be located in the deep retinal layers [53,54,55,56,57].

Rizzo et al. evaluated FTMHs before surgery by means of OCTA and en face OCT, detecting the most important vascular alterations in the DCP [55]. The authors found at this level small and circular hyporeflective cystoid cavities surrounding the FTMH in the inner nuclear layer and elongated radial hyporeflective cavities, forming a stellar pattern in the outer plexiform/Henle fiber layer complex. They described the retina surrounding the cystoid spaces as involved in a “vascular sliding” at the border of the cavities, with a residual flow surrounding the cystoid spaces, suggesting the presence of nonischemic tissue. The authors hypothesized that this phenomenon could result in an abnormal vascular structure and, consequently, in functional retinal damage. The authors also found a correlation between OCTA of the foveal region in FTMHs and en face OCT scans due to the distribution of the Müller cells in the macula.

Michalewska et al. evaluated OCTA images in FTMHs, before and after surgery [53]. The authors reported that blood flow in the deep retinal layers and choriocapillaris might be altered in FTMHs, both before and after successful surgery. The authors hypothesized that the decreased density of vessels around the macular hole in the deep retinal vessels might explain an incomplete functional recovery in some cases, especially in long-standing macular holes. They also described a “jellyfish-like shape”, sometimes visible at the level of the deep retinal vessels in FTHMs with large parafoveal cystoid spaces, suggesting that some perfusion exists around the macular hole, even if the vessels are pushed by the cystoid spaces.

Savastano et al. found a significative correlation between the BCVA and the vessel density both in the superficial and in the deep vascular plexuses at baseline and 6 months after surgery, with the most significant correlation at 6 months follow-up in the deep capillary plexus [54].

The finding that FTMH surgery may lead to an improvement in VA and to the restoration of the vessel density in deep capillary plexus corroborates the importance of vascular deep plexus impairment as a prognostic factor. Pierro et al. also observed in eyes with FTMH that DCP was the most involved capillary plexus [56]. The authors conversely found a higher vascular density in DCP than in healthy controls. Still, they attributed this finding to the possible presence of vascular engorgement, probably due to the tangential tractional forces and the steepening of the FTMH edges.

Some studies combined OCTA with functional tests, such as microperimetry, to evaluate the correlation between retinal structure and function in patients with idiopathic FTMH before and after surgery [50,57,58,59]. This integrated evaluation may be useful to determine the function–structure correlation in FTMH before and after vitreoretinal surgery to reach a better understanding of the functional consequences induced by anatomical alterations, assessing the results more objectively and potentially adding new surgical prognostic factors.

Bacherini et al. conducted a prospective study on patients with FTMH, which combined the evaluation of OCT structural findings with functional parameters obtained by OCTA and microperimetry integration: absolute scotomas corresponding to the FTMH were observed, while rings of relative scotoma in the perilesional area were detected and correlated to perifoveal cystic spaces associated to FTMH on en face OCT and OCTA [50]. Significant correlations between the extension of cystic cavities and BCVA and between reduced retinal sensitivity at 2° and 4° diameters around the FTMH and the extension of cystic areas were also found. The authors detected on the choroidal layers of OCTA scans a circular area of a peak signal, corresponding to a window defect, due to the absence of neuroretinal tissue, that made the choriocapillaris visible, and the “vascular sliding” phenomenon, with the preserved flow in the retina surrounding the cystic spaces.

Baba et al. evaluated in FTMHs the vessel density, the RS, and the inner retinal thickness before and after surgery at the four parafoveal quadrants [57]. They found a significative correlation of the vessel densities of the SCP and DCP with the RS and the inner retinal thickness at 12 months postoperatively, with a topographical peculiarity. They detected an increase in the SCP and DCP vessel density and RS in the nasal macular area, and a reduction in the temporal quadrant at 12 months postoperatively, maybe due to the nasal shift of the posterior retina, with the retinal cells no longer in their original position after surgery [57].

Kunikata et al. evaluated retinal vessel density and RS after FTMH surgery with the superior inverted ILM flap technique [58]. They found SCP vessel density unchanged after surgery, and DCP vessel density increased at an intermediate follow-up, although it returned to baseline at 6 months follow-up. They found a RS recovery, lower in the inferior sector, suggesting that ILM peeling affected postoperative visual function.

Regarding the FAZ diameter in FTHMs, it seems to be slightly larger than the minimum diameter of the macular hole and slightly smaller than the maximum diameter of the macular hole as the size of the FTMH gradually increases from the inner retinal layers to the RPE [53].

Different studies have been conducted on FAZ evaluation before and after surgery for FTMH, but they do not report conclusive data [52,56,59,60,61,62,63]. FAZ evaluation in FTMHs seems to be more difficult in the deep than the superficial plexuses, maybe due to the presence of intraretinal cysts [56]. In a few studies, the superficial FAZ area in FTMHs before surgery is reported to be the same [60] or smaller [59] than fellow eyes. Hamzah et al. reported a larger deep FAZ area in FTMHs than in fellow eyes, and the authors attributed this finding to cystoid space formation in this layer [60].

FAZ area seems to reduce after surgery, and it seems to correlate to an improvement in postoperative BCVA [52,53,59,61,62].

Wilczyński et al. found an inverse correlation between the preoperative superficial FAZ area in FTMHs and the postoperative BCVA [52]. The authors also found a contraction in the FAZ in the postoperative period, along with the resolution of the cystic changes in the middle retina on en face scans.

Baba et al. also found a decrease in the superficial FAZ area after MH closure, and they attributed this to a centripetal movement of the foveal tissue postoperatively [57]. The authors found a significant inverse correlation between the postoperative superficial FAZ and the central foveal thickness, but they did not find significant correlations with the visual function.

Michalewska et al. found that the FAZ area decreased after surgery in the superficial but not in the deep retinal layers and that the only factor predicting final visual results was the final area of FAZ at the level of deep retina vessels plexus [61]. It might be explained by the fact that the deep retina layer plexus recovers more slowly, or it might be more severely altered by the macular hole itself.

Kim et al. also found changes in the macular vasculature in eyes after MH closure, with smaller FAZ areas and lower macular parafoveal vessel densities, compared with fellow eyes; in addition they found a correlation between the deep and superficial FAZ areas and the postoperative BCVA [62].

Tsuboi et al. conversely found that after an early postoperative reduction of the FAZ calculated in the inner retinal slab, after FTMH closure, it subsequently increased toward the normal value over time [63]. The increase in postoperative FAZ area was positively correlated with the photoreceptor recovery and VA change. For this reason, the authors affirm that FAZ enlargement may be a potential biomarker for foveal reconstruction after FTMH closure.

### 2.4. Blue-Fundus Autofluorescence

**Figure 8:**
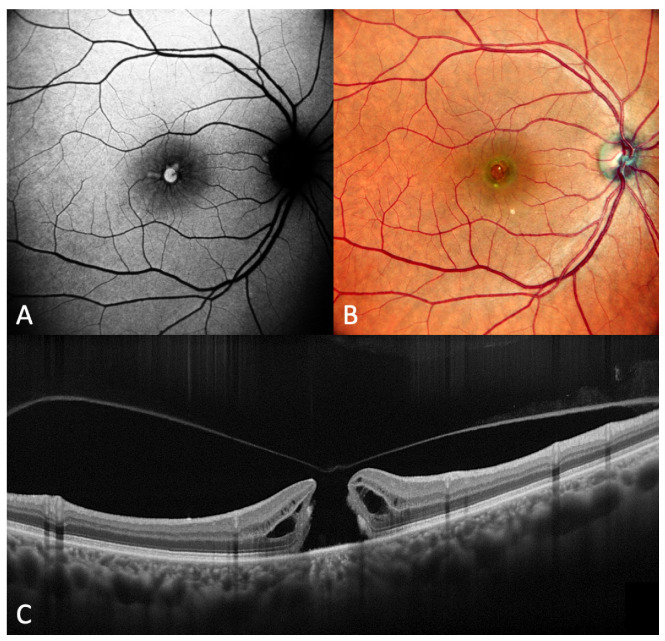
(**A**) Fundus autofluorescence (FAF) image of a full-thickness macular hole. FAF shows hyperautofluorescence in the macula. (**B**) Fundus photograph in the same eye. (**C**) Optical coherence tomography scan in the same eye.

**Figure 9:**
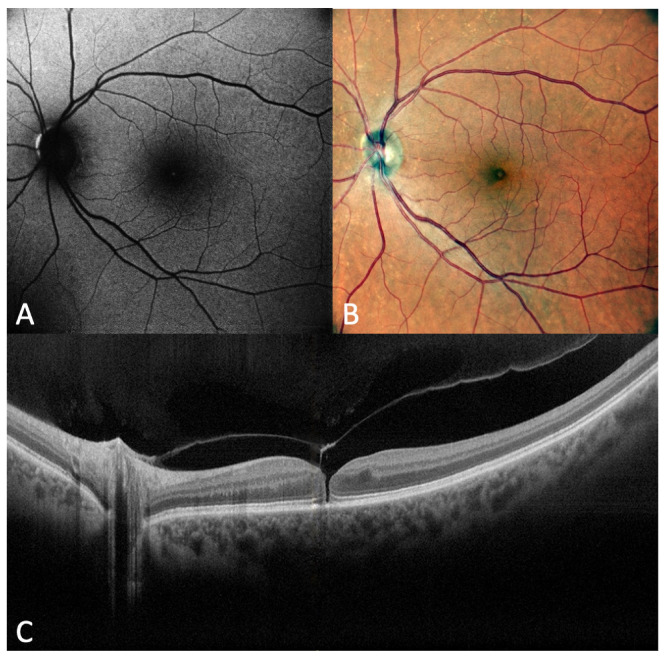
(**A**) Fundus autofluorescence (FAF) image of a small full-thickness macular hole. FAF shows a very low hyperautofluorescence in the macula. (**B**) Fundus photograph in the same eye. (**C**) Optical coherence tomography scan in the same eye.

Short-wavelength (488 nm) blue-fundus autofluorescence (B-FAF) is a non-invasive retinal imaging modality useful in the evaluation of a wide spectrum of diseases involving the retina and RPE. The short-wavelength FAF signal mainly derives from the lipofuscin in RPE cells. It can vary depending on different factors, such as the presence and quantity of absorptive pigments and structures [64,65,66]. Luteal macular pigment strongly absorbs blue light, and in a normal eye, the central accumulation of macular pigment causes a hypofluorescence central pattern on B-FAF images [67]. Abnormal B-FAF signals can be produced by changes in the amount or composition of fluorophores in RPE cells or from tissues located anteriorly [68]. In FTMH, the lack of neurosensory retina at the fovea results in an intense B-FAF signal at the site of the hole [69]. Therefore, an autofluorescent spot in the macula is consistent with a loss of the foveal tissue, either partial or complete FTMH (Figure 8 and Figure 9). The analysis of B-FAF in vitreomacular pathologies can be very useful in evaluating visual prognosis [70]. A normal FAF pattern, along with an intact ELM, should best predict good VA after surgery for idiopathic FTMH [71].

The disappearance of FAF from the FTMH, that may occur after successful surgical repair, suggests that the RPE is again covered by the retinal and/or glial tissue, as also demonstrated by the OCT images [68,72,73]. Postoperative presence of high autofluorescence in the macula conversely may indicate poor macular functional recovery and may correlate with the basal macular hole diameter, and the postoperative length of the IS/OS defect [74].

### 2.5. Adaptive Optics

**Figure 10:**
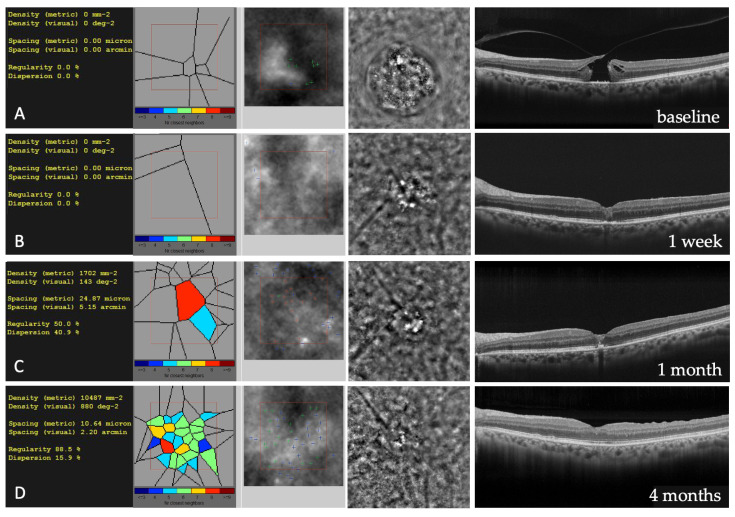
(**A**–**D**) Adaptive Optics images of cone mosaic (on the left part of the figure), with the estimation of cone density, in a full-thickness macular hole before and after surgery at different time points. Optical Coherence Tomography scans corresponding to the different adaptive optics images are depicted in the right part of the image.

Optical coherence tomography and other imaging modalities, such as scanning laser ophthalmoscopy (SLO), could fail to provide sufficiently detailed images of photoreceptor microstructure, primarily because of aberrations in ocular optics. Adaptive optics (AO) is a technology that improves image quality by reducing the effect of wavefront distortions in an optical system. In eyes in vivo, the aberrations can be compensated by using imaging systems incorporating AO, which consists of a wavefront sensor that repetitively measures aberrations on the surface of the eye and a deformable mirror or a spatial light modulator. AO integrated into retinal imaging systems, such as a flood-illuminated ophthalmoscope, SLO equipment, or fundus camera, could enhance the resolution of retinal images, and has been used to document microscopic retinal characteristics such as photoreceptors and vascular structures [75,76]. AO-flood illumination is the only commercially available AO fundus camera and enables the imaging of the retina in vivo at a cellular level. Its ultrahigh-resolution images reveal parafoveal cone photoreceptors, arteriolar texture, or pores of the lamina cribrosa in a non-invasive way [77]. The software enables the capture of the same retinal region during different visits. An automatic alignment of follow-up images allows for tracking microscopic progression or regression changes in a group of cells, a vessel section, or a lesion over time. AO has been employed in the study of photoreceptor changes after a successful FTMH closure [78,79,80]. Markan et al. conducted an AO study on three patients who underwent a successful macular hole surgery [78]. Cone density, spacing, and the number of nearest neighbors were analyzed at 2° and 4° of eccentricity in all four quadrants using AO. The study showed that there was a reduction in cell density and an increase in spacing between the cones in all the quadrants. The authors hypothesize that this is suggestive of probable migration of the cells from the parafoveal retina toward the center of the hole and that postoperative recovery of vision after successful closure of the hole occurs because of the migration or shifting of cells from the parafoveal retina toward the center, depending on the numbers of cells and their arrangement. Hansen et al. described photoreceptor structure and recovery after macular hole closure with PPV using AO scanning light ophthalmoscopy and spectral domain OCT in 4 eyes [79]. Despite successful FTMH closure, disruption of the foveal inner segment EZ was seen in all patients when imaged at 4 months after PPV. Disruption of the photoreceptor mosaic was seen using AO scanning light ophthalmoscopy at locations corresponding to regions of EZ disruption on spectral domain OCT. Cone density immediately surrounding these disruptions was normal, except for one patient. In 2 patients who were followed up serially up to 18 months after PPV, recovery of cone cells within regions of mosaic disruption could be detected over time. Caporossi et al. evaluated with AO the status of photoreceptors after failed macular hole, treated with a human amniotic membrane plug, finding hyperreflective dots at the edges of the plug that were interpreted as a mosaic of photoreceptors and some larger hypo-reflective dots in the center on the edge of the plug, perhaps corresponding to RPE or macrophagic cells [80]. AO could be useful for analyzing subtle macular features, such as photoreceptor integrity or disruption after apparent FTMH closure, and to follow up a continuous remodeling of the foveal cone mosaic after surgery (Figure 10).

**Table 1:** The role of different retinal imaging modalities in the evaluation of full-thickness macular holes.

Imaging Modality	Applications
Structural OCT	Evaluation of structural characteristics of macular holes, such as hole size, external retinal layers integrity, hole cystoid spaces, supra-RPE granular deposits, hole border morphology, and epiretinal proliferation.
Fundus Microperimetry	Functional evaluation of retinal sensitivity for the assessment of changes in macular function. It may be overlayed on OCT and OCT angiography images.
OCT Angiography	Evaluation of the retinal microvascular changes and quantitative characteristics in deep and superficial capillary plexuses. Foveal avascular zone evaluation.
Blue-Fundus Autofluorescence	Evaluation of autofluorescence pattern modifications at the site of the FTMH.
Adaptive Optics	Study of photoreceptors changes in FTMH after a successful surgical closure.

OCT: optical coherence tomography; RPE: retinal pigment epithelium; FTHM: full-thickness macular hole.

**Table 2:** Summary of key findings in full-thickness macular holes prognostic evaluation by means of different retinal imaging modalities.

Biomarker	Prognostic Value	Findings
**Structural OCT**		
Hole diameter	Negative	The minimum linear dimension of the macular hole is a critical factor for hole closure and for visual recovery after surgery [5,12,15,16,17,18,19].
ELM, EZ, and IZ defects	Negative	Preoperative length of the ELM defect was found as a strong predictor of visual acuity after FTMH surgery. The restoration of ELM, EZ, and IZ is correlated with postoperative visual recovery [22,23,24,25,26,27,28].
Intraretinal cystic changes	Unclear	Cystic spaces may affect visual acuity negatively by increasing the height of the hole edges [38]. However, cystic changes may correlate with good visual acuity after surgery if significant functional retinal tissues persist after hole closure [38].
Supra-RPE granular deposits	Negative	Supra-RPE granular deposits are a significant predictor of lower postoperative visual recovery, as they may be an indicator of photoreceptor disruption in FTMH [42].
Bumpy holes borders morphology	Negative	A bumpy morphology may suggest deeper and potentially irreversible photoreceptors damage and may negatively influence both functional and anatomical recovery [42].
Epiretinal proliferation, also known as atypical epiretinal tissue	Negative	Atypical epiretinal tissue may reflect a chronic pathogenic process involving more severe damage to the foveal tissue. FTMHs with epiretinal proliferation have worse clinical and surgical outcomes [43,44,45].
**OCTA and Microperimetry**		
Deep capillary plexus vascular alterations	Negative	In eyes with FTMHs, the main vascular alterations seem to be detected in the deep capillary plexus. An increase in deep capillary plexus after surgery is a positive prognostic factor of visual outcome [53,54,55,56,57].
Vessel density and retinal sensitivity recovery	Positive	Absolute scotomas correspond to the FTMH, while rings of relative scotoma in the perilesional area correlate to perifoveal cystic spaces associated with FTMH [50]. An increase in vessel density and retinal sensitivity in a specific topographical macular area may be due to the retinal cell shift after surgery [57].
FAZ area	Unclear	FAZ evaluation in FTMHs seems to be more difficult in the deep plexuses [56,59]. An inverse correlation seems to exist between the superficial FAZ area in FTMHs before surgery and the postoperative BCVA [52]. FAZ area appears to reduce after surgery, and it seems to correlate to an improvement in postoperative BCVA [52,53,59,61,62]. However, some authors report an early reduction after FTMH closure, and an enlargement of the FAZ over time, positively correlating with photoreceptor recovery and visual recovery [63].
**Autofluorescence**		
Intense B-FAF signal in the macula	Negative	In FTMH, the lack of neurosensory retina at the fovea results in an intense B-FAF signal at the site of the hole. Therefore, an autofluorescent spot in the macula is consistent with a loss of the foveal tissue, either partial or complete FTMH. The disappearance of fundus autofluorescence from the FTMH after successful surgical repair suggests that the RPE is again covered by the retinal and/or glial tissue [69].
**Adaptive Optics**		
Cone foveal density restoration after FTMH surgery	Positive	Adaptive optics could be useful for analyzing subtle macular features, such as photoreceptor integrity or disruption after apparent FTMH closure, and to follow up a continuous remodeling of the foveal cone mosaic after surgery [78,79,80].

OCT: Optical Coherence Tomography; ELM: External limiting membrane; EZ: ellipsoid zone; IZ: interdigitation zone; FTHM: full-thickness macular hole; RPE: retinal pigment epithelium; OCTA: Optical Coherence Tomography Angiography; FAZ: foveal avascular zone; BCVA: best-corrected visual acuity; B-FAF: blue-fundus autofluorescence.

## 3. Discussion

The rapid advances in retinal imaging over the recent decades have led to a better characterization of FTMHs and significant improvements in their diagnosis and management. The search for biomarkers predictive of surgical outcomes in this pathology is a fast-evolving field of ophthalmological research.

A large number of non-invasive tools for retinal imaging are currently available, allowing the acquisition of useful morphological and functional information. These imaging modalities include OCT, OCTA, MP, FAF, and AO. Each single imaging modality provides different and sometimes unique information and has intrinsic advantages and limitations. Recently, aiming to overcome the limitations of each single imaging modality, a multimodal imaging approach have gained popularity in the study of different retinal pathologies, both of medical and surgical interest. The combination of different retinal imaging modalities provides complementary information (structural and functional), increasing the diagnostic sensitivity and specificity and improving the comprehension of pathophysiological aspects of the diseases. Thus, the application of a different imaging approach could enhance the evaluation and management of vitreomacular interface pathologies, including FTMHs, providing a better characterization of their pre- and postoperative features. Moreover, the non-invasive retinal imaging can be periodically repeated after surgery, evaluating the morpho-functional aspects which can correlate with the final visual outcomes.

For vitreoretinal surgeons, identifying reliable morpho-functional biomarkers is meaningful for predicting surgical outcomes and choosing the best therapeutic approach. A preoperative evaluation with multiple imaging modalities may add useful information if properly interpreted.

In recent years several potential prognostic biomarkers in FTMHs have been identified by means of various non-invasive structural and functional retinal imaging techniques. In this review, we have summarized the key findings of OCT, OCTA, MP, FAF, and AO imaging in FTMHs characterization. From our point of view, structural OCT biomarkers are the most useful in clinical practice: their interpretation is direct, and the availability of this imaging modality is high. However, a combined analysis of structural OCT, OCTA, and MP overlap could be useful for a more comprehensive understanding of the functional consequences induced by anatomical alterations and for the research of new prognostic biomarkers. A few studies recently evaluated the employment of combined microperimetry and OCTA in FTHM, providing useful information on the correlation between structure and function before and after vitreoretinal surgery. However, conclusive, reliable preoperative prognostic biomarkers identifiable with OCTA are still not available. Moreover, despite the high quality of retinal vascular imaging, OCTA images are still not of simple and immediate interpretation for clinicians, and the detection of retinal vascular details information is often inaccurate, probably due to their recent introduction in clinical practice and the scarce availability of reliable biomarkers.

In this perspective, the near future directions aim to the application in clinical practice of an advanced imaging evaluation, possibly based on artificial intelligence-based image analysis, in order to achieve higher accuracy and sensitivity in detecting retinal details information. In particular, promising artificial intelligence-based models have been recently developed in ophthalmology for the prediction of different retinal pathologies, including FTMH. Obata et al. investigated if postoperative VA after FTMH surgery could be predicted by means of deep learning using preoperative OCT images [81]. The authors found that postoperative VA after MH treatment could be predicted via deep learning using preoperative OCT images with greater accuracy if compared to a model for postoperative VA prediction based on multivariate linear regression of preoperative VA, MH size, and age. Rizzo et al. explored the application of deep learning using preoperative OCTA images for the identification of morphological predictors for VA recovery in eyes affected by FTMH [82]. The authors found that the combination of preoperative superficial and deep vascular plexus datasets provided a significant morphological predictive performance for VA recovery. The application in the clinical setting of these deep learning models, based on the analysis of preoperative OCT and OCTA images, represents a future perspective in predicting functional outcomes of macular hole surgery.

## 4. Conclusions

Despite successful surgery, predicting the visual outcomes in FTMH is often difficult, and vitreoretinal surgeons need to identify the most reliable prognostic biomarkers. In this review, we resumed different preoperative factors influencing the final functional outcome, identified with different imaging modalities. We believe that the employment of different imaging modalities, if properly interpreted, could improve the comprehension of the functional effects of morphological alterations and could contribute to find new biomarkers of prognosis after surgery. In a future perspective, the application of artificial intelligence-based models in the clinical setting could support the prediction of functional outcomes in macular hole surgery.

## Data Availability

Not applicable.
